# Oxidative stress and gut-derived lipopolysaccharides in children affected by paediatric autoimmune neuropsychiatric disorders associated with streptococcal infections

**DOI:** 10.1186/s12887-020-02026-8

**Published:** 2020-03-18

**Authors:** Lorenzo Loffredo, Alberto Spalice, Francesca Salvatori, Giovanna De Castro, Cristiana Alessia Guido, Anna Maria Zicari, Paolo Ciacci, Simona Battaglia, Giulia Brindisi, Evaristo Ettorre, Cristina Nocella, Guglielmo Salvatori, Marzia Duse, Francesco Violi, Roberto Carnevale

**Affiliations:** 1 Department of Clinical, Internal, Anaesthetic and Cardiovascular Sciences, Sapienza University of Rome, I Clinica Medica, Viale del Policlinico 155, 00161 Rome, Italy; 2 Department of Pediatrics, Sapienza University of Rome, Rome, 00161 Italy; 3 Department of Medical-Surgical Sciences and Biotechnologies, Sapienza University of Rome, Latina, Italy; 4 Mediterranea Cardiocentro, Naples, Italy; 5 Neonatal Intensive Care Unit, Bambino Gesù Pediatric Hospital, Rome, Italy

**Keywords:** PANS, PANDAS, NOX2, NADPH oxidase, Oxidative stress, LPS

## Abstract

**Background:**

Paediatric autoimmune neuropsychiatric disorders associated with streptococcal infections syndrome (PANDAS) identifies patients with acute onset of obsessive-compulsive and tic disorders. The objective of this study was to evaluate serum NOX2 levels, as well as 8-iso-prostaglandin F2α (8-iso-PGF2α) and lipopolysaccharide (LPS) of PANDAS patients.

**Methods:**

In this study we wanted to compare serum levels of soluble NOX2-dp (sNOX-2-dp), iso-PGF2α and LPS in 60 consecutive subjects, including 30 children affected by PANDAS and 30 controls (CT) matched for age and gender. Serum zonulin was used as intestinal permeability assay.

**Results:**

Compared with CT, PANDAS children had increased serum levels of sNOX-2-dp, 8-iso-PGF2α and LPS. Bivariate analysis showed that serum sNOX2-dp was significantly correlated with LPS (Rs = 0.359; *p* = 0.005), zonulin (Rs = 0.444; *p* < 0.001) and 8-iso-PGF2α (Rs = 0.704; *p* < 0.001). Serum LPS significantly correlated with zonulin (Rs = 0.610; *p* < 0.001), and 8-iso-PGF2α (Rs = 0.591; *p* = 0.001). Finally, a multiple linear regression analysis showed that serum 8-iso-PGF2α and zonulin were the only independent variables associated with sNOX2-dp (R^2^ = 68%).

**Conclusion:**

This study shows that children affected by PANDAS have high circulating levels of sNOX2-dp, isoprostanes and of LPS that could be involved in the process of neuroinflammation.

## Background

Paediatric acute-onset neuropsychiatric syndrome (PANS) is defined as a wide spectrum of disorders characterised by sudden onset of obsessive-compulsive disorder (OCD) or severely restricted food intake in children [1–3]. A particular subtype of PANS is considered the paediatric autoimmune neuropsychiatric disorders associated with streptococcal infections syndrome (PANDAS) that identifies patients with acute onset of obsessive-compulsive and/or tic disorders related to Group-A streptococcus (GAS) infection [2, 4]. The close relation with streptococcus infection led to hypothesize to an autoimmune pathogenesis of PANDAS in which streptococcal antibodies cross-react with neuronal antigens [5]. This latter elicits dysregulation of dopamine receptors placed in the basal ganglia and in other types of neurons in the cortex [6] and to a persisting neuroinflammation [7].

Growing evidence showed that oxidative stress has an important role in the neuroinflammation process as shown in neurodegenerative disease and in psychotic disorders [8, 9]. Previous experimental studies found that reactive oxygen species (ROS), derived from NADPH oxidase-2 (NOX2), have a pivotal role in apoptotic pathways and in mediating the inflammatory processes in the central nervous system [10, 11]. To the best of our knowledge, no study has analyzed oxidative stress and NADPH oxidase activation in children affected by PANDAS.

Several studies in animals and humans suggested that alterations of gut microbiota are associated to neuro-inflammation [12, 13]. A recent study by Quagliarello et al. showed that children affected by PANDAS have changes of the gut microbiota that could favor the neuro-inflammation [14].

Lipopolysaccharide (LPS), derived from gram-negative bacteria, plays a pivotal role in causing neuroinflammation by an increase of oxidative stress [12, 15, 16]. A relationship among LPS, oxidative stress and NOX2 activation, in other clinical situations as NAFLD [17], pneumonia [18] atherosclerosis [19] and neurodegenerative disease [16], has been previously reported. We speculated that children affected by PANDAS have NOX2 over-activation and increased oxidative stress that may favor to onset and persistence of the disease. Thus, this study wanted to assess NOX2 and 8-iso-prostaglandin F2α (8-iso-PGF2α), as markers of oxidative stress, in serum of PANDAS and controls. Furthermore, we wanted to assess the potential role for gut-derived LPS in eliciting systemic NOX2 levels in children affected by PANDAS.

## Methods

Thirty consecutive subjects (24 males and 6 females, mean age 9 ± 3), who were referred to the Allergology and Pediatric Neurology clinic of “Sapienza” University of Rome from January 2018 to December 2019, were enrolled. Thirty control subjects (24 males and 6 females, mean age 9 ± 3), matched for aged and gender, were enrolled at the same pediatric clinic at the same period. Controls were recruited through a screening program in childhood.

Inclusion criteria were represented by: subjects aged between 3 and 16 years affected by PANDAS.

PANDAS was defined according to the criteria elaborated by Dr. Swedo and collaborators [2, 3]:

1. presence of OCD (diagnosed according to DSM IV criteria) and/or tic disorders.
2. onset of symptoms between 3 years and puberty.
3. episodic course of the disease.
4. symptoms and exacerbations temporally associated with GAS infections.
5. association with neurological anomalies (choreiform movements and motor hyperactivity during symptoms exacerbations).

Exclusion criteria were represented by: PANS not related to GAS, Sydenham corea, Tourette syndrome, Autoimmune encephalitis, Systemic autoimmune diseases, Wilson’s disease, congenital heart disease, renal disease, cancer, treatment with immuno-suppressive drugs or antioxidants, liver disease, acute disease.

The study conformed to the ethical guidelines of the 1975 Declaration of Helsinki and was approved by the Sapienza University of Rome Ethics Committee (n. 5377).

### Blood sampling

Blood samples were collected, between 8.00 and 9.00 am, in Vacutainers (Vacutainer Systems, Belliver Industrial Estate, Plymouth, UK) after an overnight fast (12 h). Samples were centrifuged at 300 g for 10 min, and the supernatant was collected and stored at − 80 °C until dosage.

### ELISA detection of sNOX2-dp

Serum NOX2 levels were measured as soluble NOX2-derived peptide (sNOX2-dp) with an ELISA method as previously reported [20]. Briefly, reference standards of sNOX2-dp and serum samples were prepared and the wells of a microtiter plate were coated overnight at 4 °C. After washing and blocking the remaining protein-binding sites, a specific anti-sNOX2dp-horseradish peroxidase (HRP) monoclonal antibody against the amino acidic sequence of the extra membrane portion of NOX2 was added in each well. Finally, after the addition of the substrate 3,3′,5,5′-tetramethylbenzidine (TMB, Bethyl Laboratories, TX, USA) and the stop solution, the absorbance of each well was read spectrophotometrically at 450 nm with a plate reader. Values were expressed as pg/ml; intra-assay and inter-assay coefficients of variation were 8.95 and 9.01%, respectively.

### 8-iso-PGF2α

8-iso-PGF2α levels were measured in serum by using a colorimetric assay kit (DRG International, Inc). Values were expressed as pmol/L. Intra-assay and inter-assay coefficients of variation were 5.8 and 5.0%, respectively.

### Serum zonulin

Serum zonulin levels were measured with a commercially ELISA kit (Elabscience). Briefly, standards and samples were added to a pre-coated microplate with a specific antibody for zonulin and incubated 90 min at 37 °C. Then, a biotinylated detection antibody and Avidin-Horseradish Peroxidase (HRP) conjugate were added to each well. The amount of zonulin was measured at a wavelength of 450 nm with a microplate auto-reader. Values were expressed as ng/ml; both intra-assay and inter-assay coefficients of variation were within 10%.

### LPS

Samples were thawed only once and used to perform specific sandwich enzyme-linked immunosorbent assay (ELISA) to measure LPS (Cusabio, Wuhan, China). The standards and samples were plated into a micro-plate pre-coated with the antibody specific for LPS. After incubation, samples were read at 450 nm. Values were expressed as pg/ml; intra-assay and inter-assay coefficients of variation were < 10%.

### Statistical analysis

Statistical analysis was performed with SPSS 18.0 software for Windows (SPSS, Chicago, IL, USA). The Kolmogorov-Smirnov test was used to determine whether variables were normally distributed. Normally distributed data are described as means±standard deviations (SDs). Group differences were analyzed by Kruskal-Wallis tests (for non-normally distributed data) or analysis of variance (ANOVA). Differences between categorical variables were assessed by the χ2 test. Simple linear regression analysis was performed by *Spearman’s rank* correlation test; the variables with evidence of an association *p* < 0.10 were included in a multivariable linear regression using an automated procedure. A *p* value < 0.05 was considered as statistically significant.

### Sample size determination

The minimum sample size was computed with respect to a two-tailed, one-sample Student t test considering, on the basis of data from a previous pilot study (data not shown): a difference of 4 pg/ml for sNOX2dp levels between children affected by PANDAS and controls, 4.7 as SD, 0.05 (α) as type I error probability and 0.95 as power 1 − β. The sample size was *n* = 30 patients/group.

## Results

**Fig. 1:**
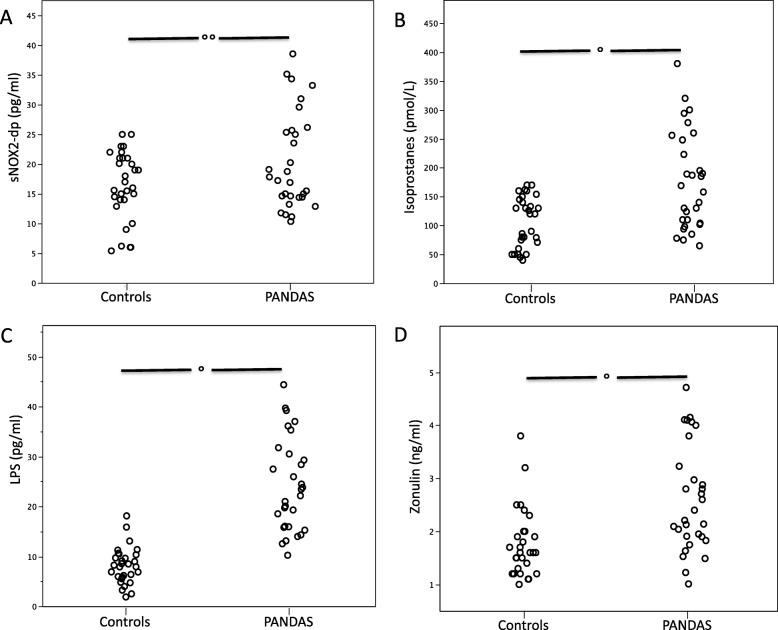
sNOX2-dp (**a**), isoprostanes (**b**), LPS (**c**) and zonulin (**d**) in PANDAS and controls. *p < 0.001; **p < 0.05.

Clinical characteristics of patients with PANDAS and controls are showed in the Table 1. No significant difference between PANDAS and controls was found for age, fasting blood glucose, blood pressure and BMI (Table 1). Conversely serum sNOX2-dp, 8-iso-PGF2α, LPS and zonulin were higher in PANDAS compared to controls (Table 1 and Fig. 1, panels a-d).

**Table 1:** Clinical and laboratory characteristics of PANDAS and controls

	PANDAS (***n*** = 30)	Controls (***n*** = 30)	***p*** value
**Age**	9 ± 3	9 ± 3	0.949
**Gender (male/female)**	24/6	24/6	1
**Glycaemia (mg/dL)**	83 ± 3.75	87 ± 3	0.617
**Systolic blood pressure (mmHg)**	101 ± 3.79	112 ± 3	0.198
**Diastolic blood pressure (mmHg)**	67 ± 2.55	70 ± 2	0.6
**BMI**	18 ± 2	17 ± 1	0.157
**Tic disorders (presence/absence)**	25/5	0	–
**OCD (presence/ absence)**	10/20	0	–
**Anti-streptolisinic title (UI/mL)**	409 ± 262	0	–
**LPS (pg/ml)**	24.1 ± 9.2	8.1 ± 3.6	< 0.001
**sNOX2-dp (pg/ml)**	20.4 ± 8.1	16.3 ± 5.7	0.02
**Zonulin (ng/ml)**	2.6 ± 1	1.7 ± 0.6	0.005
**8-iso-PGF2α** **(pmol/L)**	175 ± 84	106 ± 43	0.001

Bivariate analysis showed that serum sNOX2-dp was significantly correlated with 8-iso-PGF2α (Rs = 0.704; *p* < 0.001), LPS (Rs = 0.359; *p* = 0.005) and zonulin (Rs = 0.444; *p* < 0.001). The same statistical analysis showed that serum LPS significantly correlated with 8-iso-PGF2α (Rs = 0.591; *p* < 0.001) and zonulin (Rs = 0.610; *p* < 0.001). Furthermore, serum 8-iso-PGF2α correlated with the tic disorders (Rs = 0.382; *p* = 0.03).

Multiple linear regression analyses, showed that serum 8-iso-PGF2α (SE: 0.011; standardized coefficient β: 0.780; *p* < 0.001) and zonulin (SE: 0.911; standardized coefficient β: 0.241; *P* = 0.04) were the only independent predictive variables associated with sNOX2-dp (R^2^ = 68%).

## Discussion

This study reports that patients with PANDAS have high sNOX-2-dp levels and suggests a potential role for gut microbiota as a source of oxidative stress in this population.

NOX-2 derived oxidative stress leads to inflammation in several neurologic diseases as Amyotrophic lateral sclerosis, Parkinson’s disease and Alzheimer’s disease [10, 16]. Furthermore, NOX2 activation seems to be involved in the pathogenesis of psychotic disorders, as schizophrenia, leading to an imbalance of excitation and inhibition in cortical neural circuits [21]. To the best of our knowledge, NOX2 activation and oxidative stress has never been studied in patients with PANS and PANDAS. This study reports that PANDAS subjects have high levels of sNOX2-dp and high levels of isoprostanes, suggesting an increased systemic oxidative stress derived from NOX2 activation in this neuropsychiatric disorder. Another interesting result of this study is the positive correlation between TIC and isoprostanes, hypothesizing a direct relationship between oxidative stress and neuropsychiatric manifestation in PANDAS.

Previous studies identified dysbiosis in patients suffering from neurologic diseases and proposed the concept of “gut-brain-axis” as source of neuroinflammation [22–24]. Recently, Quagliarello et al. showed that children affected by PANDAS also have gut dysbiosis [14]. Furthermore, the same authors hypothesized that streptococcal infections alter gut microbiota and consequently lead to a proinflammatory state in the gut by selection of specific bacterial strains [14]. Gram negative bacteria of gastro-intestinal tract secrete LPS that exerts pro-inflammatory actions on neurons [15]. Animal studies showed that LPS increases neuroinflammation by NOX2 activation [15, 25, 26]; however, the mechanism through which LPS damages the nervous system is unclear.

LPS has been hypothesized to have a pathogenetic role in PANDAS [27], although no study evaluated LPS serum levels in this neuropsychiatric disorder. Thus, to address this issue we studied LPS levels in PANDAS. We found that subjects affected by PANDAS disease have higher LPS levels that are linearly associated with sNOX2-dp levels and with isoprostanes. This association suggests a link between LPS and oxidative stress in PANDAS. However, further studies are needed to establish the pathophysiological mechanisms responsible for neuroinflammatory process.

To evaluate whether gut permeability can explain the LPS increase in PANDAS, we assessed the circulating levels of zonulin, which modulate gut permeability by disassembling the intercellular tight junctions [28]. Previous studies showed that zonulin up-regulation increases gut permeability [29]. The high serum levels of zonulin in PANDAS patients and its correlation with serum LPS provide the evidence that gut permeability is enhanced in this neuropsychiatric disorder and may be responsible for the increased circulating levels of LPS.

The study has some limitations. NOX2 and oxidative stress were studied in blood and not in the nervous system by biopsies. However, this procedure is unethical and invasive. Furthermore, we did not assess other NADPH isoforms, such as NOX1 and NOX4, which could also increase oxidative stress. The present study did not address the mechanism that contribute to the translocation of LPS from the gut microbiota to the central nervous system. However, changes in intestinal permeability may be a plausible mechanism, since an increase in serum zonulin is significantly correlated with blood LPS.

## Conclusions

This study shows that children affected by PANDAS have high circulating levels of sNOX2-dp, isoprostanes and of LPS that could be potentially implicated in the process of neuroinflammation.

## Data Availability

The data used to support the findings of this study are available from the corresponding author upon request.

## Abbreviations

PANS: Pediatric acute-onset neuropsychiatric syndrome
PANDAS: Pediatric autoimmune neuropsychiatric disorders associated with streptococcal infections syndrome
NADPH oxidase: Nicotinamide-adenine dinucleotide phosphate oxidase
8-iso-PGF2α: 8-iso-prostaglandin F2α
LPS: Lipopolysaccharide
OCD: Obsessive-compulsive disorder
GAS: Group-A streptococcus
